# Spatial and temporal influences on the physiological condition of invasive silver carp

**DOI:** 10.1093/conphys/cot017

**Published:** 2013-07-11

**Authors:** Stephanie A. Liss, Greg G. Sass, Cory D. Suski

**Affiliations:** 1 Department of Natural Resources and Environmental Sciences, University of Illinois, Urbana, IL 61801, USA; 2 Wisconsin Department of Natural Resources, Boulder Junction, WI 54512, USA; 3 Illinois Natural History Survey, University of Illinois, Champaign, IL 61820, USA

**Keywords:** Invasive species, landscape, macrophysiology, nutrition, stress

## Abstract

We quantified nutritional and stress parameters (alkaline phosphatase, cholesterol, protein, triglycerides, cortisol, and glucose) in invasive silver carp (*Hypophthalmichthys molitrix*) inhabiting four large rivers throughout three distinct time periods in the Midwestern USA. Examining the basic biology and ecology of an invasive species is crucial to gain an understanding of the interaction between an organism and its environment. Analysis of the physiological condition of wild-caught silver carp across broad spatial and temporal scales is essential because stress and nutritional parameters can link individuals to their habitats and vary among populations across environments. During each time period, we collected blood samples from individual silver carp in the Illinois River and portions of the Mississippi, Ohio, and Wabash rivers in Illinois. We tested for relationships between silver carp nutrition and stress across rivers, reaches within rivers, and time periods. Principal component analyses separated physiological parameters into a stress component (cortisol and glucose) and two nutritional components representative of short-term feeding (alkaline phosphatase, protein, and triglycerides) and body energy reserves (cholesterol and protein). Akaike's information criterion suggested that time period had the greatest influence on stress. Stress levels were consistent in all four rivers, and declined across time periods. Akaike's information criterion also suggested that interactions of time period and river had the greatest influence on short-term feeding and body energy reserves. There was no specific pattern across time periods within each river, nor was there a pattern across rivers. Our results provide a better understanding of nutritional and stress conditions in invasive silver carp across a broad landscape and temporal scale, with implications for managing and predicting the spread of this species.

## Abstract (precis)

This study quantified the nutritional and stress physiology of invasive silver carp across broad spatial and temporal scales to define how these fish interact with their environment. Results demonstrated that nutritional properties varied significantly across rivers and sampling time. Stress levels varied only across time periods.

## Introduction

Riverine ecosystems are spatially and temporally dynamic, and this variability may influence habitat quality for fishes. For example, hydrological regimes, nutrient loading, seasonal variation, and human activity can interact, or act independently, to generate lotic habitat characteristics that vary across an extreme range of conditions (Karr *et al.*, 1985; Pyron and Neumann, 2008; McClelland *et al.*, 2012). Water temperature may vary spatially or seasonally along environmental gradients caused by channel morphology and location within the river, which may influence the density, diversity, and/or richness of aquatic communities (Schlosser, 1990). Warmer water temperatures may induce growth or spawning (Bayley, 1995; DeGrandchamp *et al.*, 2007) and increase feeding rates (Kitchell *et al.*, 1977), while cooler temperatures may lead to decreased feeding and lower metabolic rates (Bohl, 1980). Seasonal variation in flow rates can cause lotic environments to vary from slow to fast moving, which may also provide suitable environmental conditions for larval production in fish (Lohmeyer and Garvey, 2008). In addition to the influence of broad-scale factors, habitat parameters that operate at smaller scales can also influence resident fish and aquatic communities. Competition for resources (e.g. food availability, thermal habitat, and refuge) may occur within high-quality lotic habitats, with a competitive advantage offered to individuals that obtain access to these preferred habitats (Huey, 1991).

Access to habitat of varying quality may influence decisions by individual fish that relate to reproduction and/or movement, which may ultimately influence abundance, distribution, and mortality. Resource allocation is often shaped by habitat quality, with high-quality habitat providing sufficient food accessibility (maximizing fitness) and low-quality habitat potentially forcing an animal to disperse and find sufficient resources (Huey, 1991; Homyack, 2010). For example, the energy required for optimal growth and recruitment can be influenced by available resources, including access to food, nutritional status, and the energy expenditure of parents (Encina and Granado-Lorencio, 1997; Homyack, 2010). Likewise, variation in thermal regimes may reduce foraging activity (Burel *et al.*, 1996) or prey availability (Wahl *et al.*, 2008; Burdis and Hoxmeier, 2011), which may restrict food access. Related to habitat quality is the notion of allostasis, and the influence that habitat quality can have on allostatic load, which, in turn, can influence abundance, distribution, and mortality. Essentially, individuals strive to maintain a balance between daily and seasonal environmental variation, and unpredictable events that can affect energy requirements. Higher-quality habitats may provide environmental conditions that minimize an organism's allostatic load and, in turn, can reduce energetic expenditure (McEwen and Wingfield, 2003). Thus, understanding how fish populations interact with habitat of varying quality at broad temporal and spatial scales can provide insights into population-level trends, including energy use, movement, dispersal, reproduction, and mortality.

Physiological tools are an integral way to quantify organism–environment interactions, particularly when considered over broad spatial scales (Chown *et al.*, 2004; Chown and Gaston, 2008; Homyack, 2010), and can link individuals to the habitats in which they reside (Ricklefs and Wikelski, 2002; Cooke and Suski, 2008; Ellis *et al.*, 2012). In particular, aspects of physiology that quantify the stress and nutritional status of individuals are particularly insightful. Stress and nutritional metrics represent how behaviour and physiology interact within dynamic environments to influence individual homeostasis and balance energy requirements that are not reflected in simply quantifying habitat occupancy (Ellis *et al.*, 2012; Raubenheimer *et al.*, 2012). More importantly, changes at the population level (e.g. abundance and distribution) occur in response to changes in individuals (Ricklefs and Wikelski, 2002; Ellis *et al.*, 2012), making the quantification of animal health, stress, and condition through blood chemistry valuable for understanding links between physiology, community structure, competition, and movement.

Silver carp (*Hypophthalmichthys molitrix*) are relatively recent invaders to North America (early 1970s; Chick and Pegg, 2001; Kolar *et al.*, 2007) and represent an excellent model organism for asking questions regarding the impacts of broad- and small-scale habitat variation on the stress, condition, and distribution of wild organisms. Silver carp have exhibited rapid population growth and spread since their introduction, making them the dominant species in several riverine ecosystems in the Midwestern USA (Kolar *et al.*, 2007; Sass *et al.*, 2010). More importantly, silver carp can have multiple spawning bouts per year, have few natural predators as adults, and feed low on the food chain (primarily prioritizing planktonic prey), all of which may be responsible for their success as invaders (Chick and Pegg, 2001; Kolar *et al.*, 2007). Currently, we do not know how various broad- and small-scale parameters influence the health and condition of silver carp, or how these different factors can influence the movement, activity, and spread of this invasive species. Information on stress and condition at broad scales can have important implications for understanding not only how organisms interact with their environments, but also which factors influence their spread and distribution across the landscape. Therefore, our objective was to quantify variation in blood-based nutritional and stress parameters of silver carp across broad spatial and temporal scales.

## Materials and methods

### Field analysis

**Figure 1:**
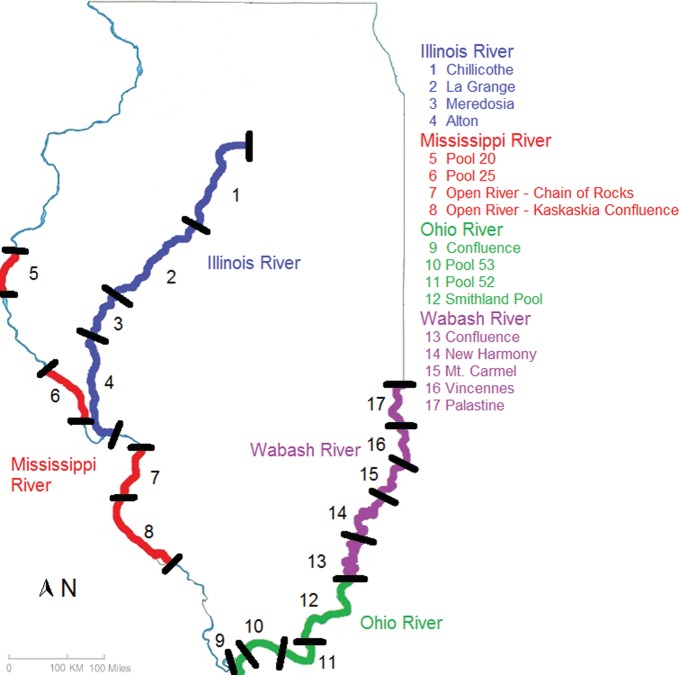
Map of the Illinois River and portions of the Mississippi, Ohio, and Wabash rivers in Illinois, illustrating reaches sampled in 2011. The Illinois River consists of the Alton reach [river mile (RM) 0–70], Meredosia (RM 70–80), La Grange (RM 80–158), and Chillicothe (RM 158–231). The Mississippi River consists of Pool 20 (RM 343–364.5), Pool 25 (RM 242–273.5), Chain of Rocks (RM 165.5–200.5) and Kaskaskia (RM 117–165.5). The Ohio River consists of Smithland Pool (RM 848–918.5), Pool 52 (918.5–939), Pool 53 (RM 939–962.5), and the Confluence (RM 962.5-981). The Wabash River consists of Terre Haute (RM 315.5–351), Palestine (RM 351–385.5), Vincennes (RM 385.5–412), Mt Carmel (RM 412–444.5), and New Harmony (RM 444.5–487).

We collected fish in association with two long-term fish-monitoring programmes co-ordinated by the Illinois River Biological Station: the Long-Term Resource Monitoring Program (LTRMP) and the Long-Term Illinois, Mississippi, Ohio, and Wabash River Fish Population Monitoring Program (LTEF; Gutreuter *et al.*, 1995; Tyszko *et al.*, 2012). Ancillary water quality data, including Secchi disk transparency, water temperature, dissolved oxygen, surface velocity, and river stage, were also collected during fish sampling. Water temperature and dissolved oxygen were collected with a hand-held YSI (model 85), and velocity was measured with an Acoustic Doppler Velocimeter (ADV), according to LTEF sampling protocols (Tyszko *et al.*, 2012). River stage information was obtained from the US Army Corps of Engineers river gauge readings (http://rivergages.mvr.usace.army.mil/WaterControl/new/layout.cfm). We selected four reaches with established populations of silver carp in each of the Illinois, Mississippi, and Ohio rivers, and five reaches within the Wabash River for fish collection. These four rivers are all part of the Mississippi River Basin and have extensive agricultural land use, which has increased nutrient levels basin-wide (Donner, 2003). Reaches sampled in the Illinois River included Alton, Chillicothe, La Grange, and Meredosia (Fig. 1). In the Mississippi River, reaches consisted of Chain of Rocks, Kaskaskia, Pool 20, and Pool 25 (Fig. 1). The Confluence, Pool 52, Pool 53, and Smithland were sampled in the Ohio River, and Mount Caramel, New Harmony, Palestine, Terre Haute, and Vincennes reaches were our sites in the Wabash River (Fig. 1). Sampling was divided into three time periods (mid-summer = 15 June –31 July; late summer = 1 August–15 September; and early autumn = 16 September–31 October; Table 1). We sampled blood from up to 14 silver carp in each selected reach from the four rivers during each time period (Table 1).

**Table 1:** Silver carp (*Hypophthalmichthys molitrix*) sampling at broad temporal and spatial scales across mid-summer, late summer, and early autumn time periods in the Illinois, Mississippi, Ohio, and Wabash rivers during 2011

Time period	Sampling dates	River	Number of reaches	Total no. of fish sampled per river per time period
Mid-summer	15 June–31 July 2011	Illinois	4	53
		Mississippi	4	47
		Ohio	4	51
		Wabash	5	32
Late summer	1 August–14 September 2011	Illinois	3	30
		Mississippi	4	43
		Ohio	4	43
		Wabash	5	50
Early autumn	15 September–31 October 2011	Illinois	4	40
		Mississippi	4	34
		Ohio	4	40
		Wabash	5	50

We collected silver carp primarily using pulsed direct current (DC) electrofishing according to methods outlined by Gutreuter *et al.* (1995) and Tyszko *et al.* (2012). Some of the silver carp analysed leapt on board the boat prior to being stunned by electricity and were sampled for blood in a manner identical to those that were stunned by electricity and labelled accordingly. Immediately after capturing a fish, we collected blood from the caudal vessel using either a 2.5 or a 3.8 cm needle (BD PrecisionGlide needles; 22 gauge) with a 1 ml syringe (BD Slip Tip Sterile Syringes; volume 1 ml) pre-rinsed in heparin saline (Houston, 1990). We drew blood from all individuals, regardless of collection method, in <3 min to obtain a baseline value of stress not influenced by sampling procedures (Romero and Reed, 2005; Ellis *et al.*, 2012). This also qualifies as baseline nutritional status, because nutritional values have not been shown to change in <3 min (Congleton and Wagner, 2006; Gingerich *et al.*, 2010). One millilitre of whole blood was collected, placed into a microcentrifuge tube, and spun at 5200 *g* for at least 3 min to separate plasma from red cells. Plasma was removed using a transfer pipette and placed into two additional 1.5 ml microcentrifuge tubes. Plasma and red cells were then flash frozen in a dry shipper charged with liquid nitrogen (Suski *et al.*, 2006). Samples were transported to the University of Illinois at Urbana-Champaign and stored in a <− 75°C freezer until processing. We also recorded total length (in millimetres) and weight (in grams) for each fish.

### Laboratory analysis

We analysed plasma triglycerides (in milligrams per decilitre), cholesterol (in milligrams per decilitre), alkaline phosphatase (ALP; in units per litre), and cortisol (in nanograms per millilitres) using the following commercially available kits: EnzyChrom Triglyceride Assay Kit (ETGA-200), EnzyChrom Cholesterol Assay Kit (ECCH-100), and QuantiChrom Alkaline Phosphatase Assay Kit (DALP-250), respectively (BioAssay Systems, Haywood, CA, USA), and Cortisol EIA Kit (ADI-900-071; Enzo Life Sciences, Plymouth Meeting, PA, USA). Plasma glucose (in milligrams per decilitre) was determined enzymatically with a microplate spectrophotometer (Spectra Max Plus 384 model #05362; Molecular Devices, Union City, CA, USA) following the procedure of Lowry and Passonneau (1972). We measured protein (in grams per decilitre) with a hand-held protein refractometer (AST model 1250; Thomas Scientific, Swedesboro, NJ, USA; Wells and Pankhurst, 1999), certified for use in the range of 0–12 g dl^−1^. The cortisol EIA kit we used has been identified as accurate and precise when used for fishes (Sink *et al.*, 2008), and has a lower detection limit of 0.0567 ng ml^−1^. Individuals below sensitivity limits were treated as being equal to the lowest detection limit value for the kit (0.0567 ng ml^−1^; Haddy and Pankhurst, 1999; Ramsay *et al.*, 2006). Cholesterol, ALP, protein, and triglycerides have been shown to represent either a short-term, recent feeding nutritional component or a long-term body energy reserve nutritional component (Wagner and Congleton, 2004; Congleton and Wagner, 2006; Guerreiro *et al.*, 2012). Cortisol and glucose are associated with the stress response of teleost fishes (Barton, 2002; Wagner and Congleton, 2004; Congleton and Wagner, 2006).

### Statistical analyses

We used a multivariate principal component analysis (PCA) to reduce the dimensionality of the data and to quantify relationships between nutritional and stress parameters of silver carp among rivers, among reaches within a river, and over time (Vega *et al.*, 1998; Smith, 2002); our sample sizes met PCA minimums recommended by Green (1991). Principal components (PCs) with eigenvalues >1 were used for analysis. Selected PCs underwent varimax factor rotation to maximize the amount of variation explained by each factor (Kaiser, 1960; Gingerich and Suski, 2011). Components with eigenvectors >0.4 or <− 0.4 contributed maximally to each principal component (Kaiser, 1960; Gingerich and Suski, 2011). Positive factor loadings (>0.0) indicated a positive correlation with the raw data, and negative factor loadings (<0.0) indicated a negative correlation with the raw data. Rotated PCs were then treated as independent response variables in subsequent analyses. The fits of predictor variables (river, reach, time period, total length, and their interactions) were compared against each PC and assessed using biologically relevant models chosen *a priori*. Reaches were nested within rivers (as denoted by ‘[]’ in Table 2) because the effects of reach can occur only within a single level of another variable (i.e. the river where the reach is located). Variables were crossed (as denoted by ‘ × ’ in Table 2) to determine interactions between variables.

**Table 2:** Model selection results relating predictor variables to variation in principal component scores for wild-caught silver carp (*H. molitrix*)

Principal component	Model	AICc	Δ AICc	AICc weight	Model likelihood
PC1	Time period[River]	1327.90	0.00	1.00	1.00
	Reach × time period[River]	1344.38	16.48	0.00	0.00
	Time period	1366.63	38.73	0.00	0.00
	Reach[River]	1380.43	52.53	0.00	0.00
	River	1405.80	77.90	0.00	0.00
	River × total length	1414.76	86.86	0.00	0.00
	Total length	1420.61	92.71	0.00	0.00
	Time period × total length	1422.73	94.83	0.00	0.00
PC2	Time period[River]	1377.57	0.00	1.00	1.00
	Time period	1389.23	11.66	0.00	0.00
	River × total length	1402.61	25.04	0.00	0.00
	River	1412.81	35.24	0.00	0.00
	Total length	1414.78	37.21	0.00	0.00
	Time period × total length	1418.32	40.75	0.00	0.00
	Reach × time period[River]	1428.21	50.64	0.00	0.00
	Reach [River]	1431.08	53.51	0.00	0.00
PC3	Time period	1383.35	0.00	1.00	0.90
	River × total length	1390.13	6.78	0.03	0.03
	River	1390.34	6.99	0.03	0.03
	Total length	1390.76	7.41	0.02	0.02
	Time period[River]	1391.08	7.73	0.02	0.02
	Time period × total length	1405.30	21.95	0.00	0.00
	Reach × time period[River]	1418.16	34.81	0.00	0.00
	Reach[River]	1421.07	37.72	0.00	0.00

Principal component (PC) scores are as follows: PC1 corresponds to a short-term feeding score; PC2 represents a body energy reserve score; and PC3 represents a stress score. Fish were collected from four rivers across three time periods and sampled for blood immediately following collection. Models are ranked by differences in Akaike's information criterion values (corrected for small sample size; ΔAICc), and the model with the lowest ΔAICc value is the best fit to the data, with AICc weight determining the best approximating model.

Competing models were compared using Akaike's information criterion corrected for small sample size (AICc) to quantify and rank the best approximating model (Grueber *et al.*, 2011; Hegyi and Garamszegi, 2011; Symonds and Moussalli, 2011). Based on the ΔAICc, a value 0 ≤ ΔAICc ≤ 2 shows substantial support that a model is the best fit to the data, while an ΔAICc >10 shows little support (Burnham and Anderson, 1998; Suski and Ridgway, 2007; Symonds and Moussalli, 2011). Following best fit model determination for each rotated PC, a mixed-model analysis of variance (ANOVA) for the best-fitting model was compared using a Tukey's multiple comparison *post hoc* analysis to aid with visualization of trends in the data (Demsar, 2006). We performed all statistical analyses using JMP version 10.0 (SAS Institute, Cary, NC, USA). Rejection of the null hypothesis (α) for all tests was 0.05. All values are reported as means ± SEM where appropriate. We used the null hypothesis of no variation in stress or nutrition (i.e. physiological parameters) for silver carp across rivers, across reaches within rivers, or across time periods.

## Results

The total length, weight, and the six nutritional and stress parameters of silver carp were highly variable among rivers and time periods (Table 3). Total length and weight were strongly correlated (*P* < 0.05, *r*^2^ = 0.90); therefore, only total length was included as a variable in analyses. Out of the 504 cortisol readings for silver carp, 297 fish had cortisol values below the detection limit of the kit (Table 3). Out of the 513 fish sampled, 110 fish leapt onto the boat. Environmental parameters were also variable among rivers and time periods (Table 4).

**Table 3:** Sample size, minimum, maximum, mean, median, and SEM values for several nutritional and stress parameters of silver carp (*H. molitrix*), sampled in the Illinois, Mississippi, Ohio, and Wabash rivers during 2011

River	Parameter	*n*	Minimum	Maximum	Mean	Median	SEM
Illinois	Triglycerides (mg dl^−1^)	118	10.1	298.1	96.8	84.5	5.0
	Cortisol (ng ml^−1^)	122	0.06	102.5	10.2	2.3	1.7
	Cholesterol (mg dl^−1^)	123	44.3	592.0	218.0	211.4	5.8
	Glucose (mg dl^−1^)	123	18.8	73.2	39.6	38.2	1.0
	Protein (g dl^−1^)	123	2.0	5.6	3.7	3.7	0.0
	ALP (U l^−1^)	123	3.0	105.2	35.2	33.1	2.0
	Weight (g)	123	160.0	3890.0	1212.6	1120.0	49.2
	Total length (mm)	123	311	709	495	487	5
Mississippi	Triglycerides (mg dl^−1^)	121	6.6	357.8	97.0	79.6	6.0
	Cortisol (ng ml^−1^)	120	0.06	288.0	12.6	0.1	3.3
	Cholesterol (mg dl^−1^)	124	57.4	557.3	190.5	182.7	6.6
	Glucose (mg dl^−1^)	124	11.0	78.6	40.0	38.1	1.3
	Protein (g dl^−1^)	124	2.0	5.3	3.5	3.5	0.1
	Alkaline phosphatase (U l^−1^)	124	5.6	142.2	25.7	21.4	1.7
	Weight (g)	124	540.0	6450.0	1905.4	1465.0	107.1
	Total length (mm)	124	387	872	599	524.5	10
Ohio	Triglycerides (mg dl^−1^)	134	4.7	527.2	109.0	77.4	7.7
	Cortisol (ng ml^−1^)	131	0.06	292.7	13.0	0.1	3.9
	Cholesterol (mg dl^−1^)	134	71.1	526.0	207.2	198.3	6.5
	Glucose (mg dl^−1^)	134	1.2	168.7	51.4	46.9	2.0
	Protein (g dl^−1^)	134	2.0	5.6	3.7	3.7	0.1
	Alkaline phosphatase (U l^−1^)	134	6.8	146.8	26.8	22.5	1.5
	Weight (g)	134	140.0	7930.0	2930.9	2760.0	146.4
	Total length (mm)	134	252	900	631	660	13
Wabash	Triglycerides (mg dl^−1^)	132	42.0	589.7	140.8	116.2	7.2
	Cortisol (ng ml^−1^)	131	0.06	97.1	9.1	0.1	1.7
	Cholesterol (mg dl^−1^)	132	79.1	369.7	168.4	164.4	3.6
	Glucose (mg dl^−1^)	132	16.5	98.7	40.7	38.3	1.2
	Protein (g dl^−1^)	132	2.9	5.6	3.9	4.0	0.0
	Alkaline phosphatase (U l^−1^)	132	7.1	111.0	25.3	20.5	1.4
	Weight (g)	132	140.0	7000.0	2952.3	2335.0	149.3
	Total length (mm)	132	262	866	615	595	12
Total	Triglycerides (mg dl^−1^)	505	4.7	589.7	111.6	90.5	3.4
	Cortisol (ng ml^−1^)	504	0.06	292.7	11.2	0.1	1.4
	Cholesterol (mg dl^−1^)	513	44.3	592.0	195.7	186.2	3.0
	Glucose (mg dl^−1^)	513	1.2	168.7	43.1	40.3	0.8
	Protein (g dl^−1^)	513	2.0	5.6	3.7	3.7	0.0
	Alkaline phosphatase (U l^−1^)	513	3.0	146.8	28.1	22.5	0.8
	Weight (g)	513	140.0	7930.0	2276.5	1810.0	69.1
	Total length (mm)	513	252	900	577	563	6

**Table 4:** Ancillary environmental data collected across rivers and time periods sampled in 2011

Time period	Environmental variable	Illinois River		Mississippi River		Ohio River		Wabash River	
		Mean	SEM	Mean	SEM	Mean	SEM	Mean	SEM
Mid-summer	Secchi depth transparency (cm)	29.09	0.43	23.68	0.93	24.49	0.61	12.00	1.03
	Surface velocity (m s^−1^)	0.54	0.03	0.49	0.04	0.37	0.04	0.75	0.09
	Water temperature (°C)	28.45	0.18	29.34	0.14	25.35	0.08	23.33	0.09
	Dissolved oxygen (mg l^−1^)	4.88	0.20	5.79	0.12	6.18	0.06	6.23	0.06
	River stage (m)	3.88	0.25	6.57	0.32	10.44	0.21	5.07	0.02
Late summer	Secchi depth transparency (cm)	28.77	1.20	29.67	1.35	44.77	1.65	31.40	0.75
	Surface velocity (m s^−1^)	0.27	0.04	0.46	0.04	0.18	0.02	0.21	0.02
	Water temperature (°C)	28.36	0.41	28.16	0.11	32.31	0.09	25.42	0.38
	Dissolved oxygen (mg l^−1^)	6.06	0.33	6.66	0.15	6.47	0.25	10.00	0.28
	River stage (m)	4.71	0.42	4.69	0.16	7.22	0.30	0.98	0.03
Early autumn	Secchi depth transparency (cm)	30.85	0.78	24.24	1.23	51.68	2.16	34.68	1.44
	Surface velocity (m s^−1^)	0.24	0.01	0.33	0.05	0.22	0.02	0.39	0.03
	Water temperature (°C)	17.47	0.52	16.89	0.33	23.54	0.07	20.42	0.21
	Dissolved oxygen (mg l^−1^)	8.04	0.10	8.51	0.06	8.57	0.11	10.07	0.17
	River stage (m)	2.79	0.24	3.73	0.31	6.55	0.28	1.26	0.04

Values shown were averaged across time periods for all reaches. All parameters were measured in real time concurrently with fish sampling, except for river stage. River stage was acquired from the Army Corp of Engineers river gauges website (http://rivergages.mvr.usace.army.mil/WaterControl/new/layout.cfm).

Principal components analysis produced three factors with eigenvalues >1, which described 71% of the total variation in the physiological parameters tested (Table 5). The first principal component (PC1) explained 28% of the variation and was characterized by positive factor loadings for triglycerides, ALP, and protein (Table 5), characteristic of short-term feeding. The second principal component (PC2) explained 25% of the variation and was characterized by positive factor loadings for cholesterol and protein (Table 5), describing long-term body energy reserves. The third principal component (PC3) explained 18% of the variation and was characterized by positive factor loadings for cortisol and glucose (Table 5), representative of stress.

**Table 5:** Principal components summarizing stress and nutritional characteristics for silver carp (*H. molitrix*) sampled from the Illinois, Mississippi, Ohio, and Wabash rivers during 2011

	PC1	PC2	PC3
Triglycerides (mg dl^−1^)	0.87^a^	0.027	−0.050
Cortisol (ng ml^−1^)	−0.12	0.10	0.87^a^
Cholesterol (mg dl^−1^)	−0.16	0.89^a^	0.066
Glucose (mg dl^−1^)	0.39	−0.28	0.64^a^
Protein (g dl^−1^)	0.43^a^	0.76^a^	−0.16
Alkaline phosphatase (U l^−1^)	0.70^a^	0.032	0.10
percentage of variance explained	28	25	18
Eigenvalue	1.7	1.5	1.1

Variables were loaded into three principal components (PC1, PC2, and PC3).

^a^Characteristics contributing maximally to each principal component are indicated and are >0.4. Positive values for each principal component correlate positively with the stress and nutritional characteristics.

**Figure 2:**
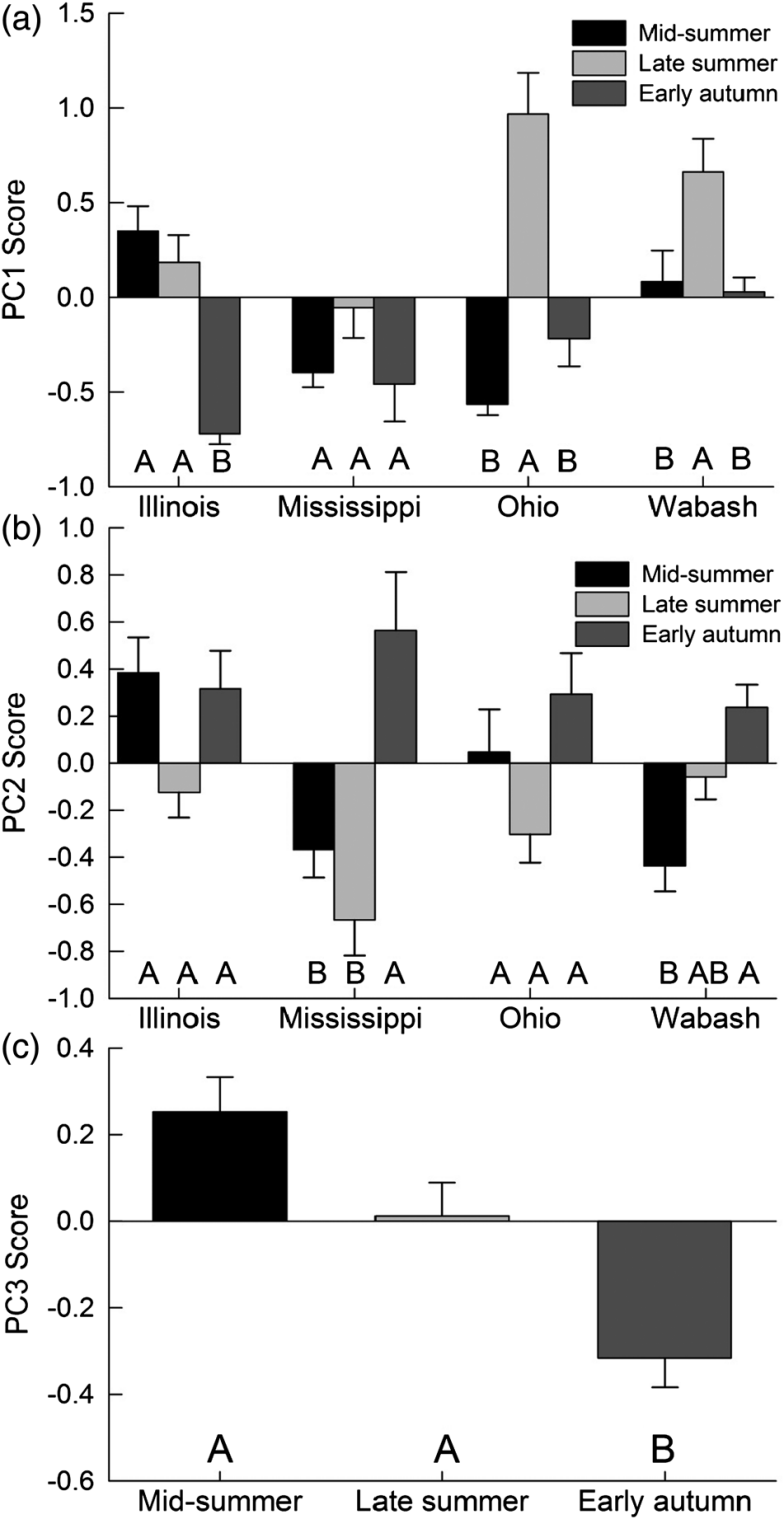
Visualization of the best fit Akaike's information criterion (corrected for small sample size; ΔAICc) ranked model linking PC1 (short-term feeding) scores (a) and PC2 (body energy reserves) scores (b) for silver carp (*Hypophthalmichthys molitrix*) by time period, with each river considered independently. PC3 (stress) scores (c) for silver carp are best explained by time period. Results of statistical analysis are reported in the figure, with dissimilar letters indicating significant differences.

Variation in PC1 was best explained by the model comprised of time period, nested within river (Table 2). The ANOVA identified significant differences in PC1 scores across each of the time periods per river [*F*_(3,_ _487)_ = 8.33, *P* < 0.0001]. *Post hoc* analysis showed that, in the late summer, silver carp PC1 scores peaked in the Ohio River, with negative short-term feeding scores in the other two time periods (Fig. 2a). Likewise, PC1 scores in the Wabash River peaked in the late summer, with lower scores in the other two sampling periods (Fig. 2a). In the Illinois River, PC1 scores in the early autumn were lowest relative to the mid-summer and late summer time periods. The PC1 scores across time periods in the Mississippi River did not differ (Fig. 2a). Variation in PC2 was also best explained by the model comprised of time period, nested within river (Table 2). The ANOVA for the best fit model of PC2 confirmed significant variation across each of the time periods per river [*F*_(3, 487)_ = 7.40, *P* < 0.0001]. *Post hoc* analysis showed that silver carp sampled from the Mississippi River had PC2 scores that peaked in the early autumn relative to sampling periods earlier in the year (Fig. 2b). Wabash River PC2 scores were also significantly greater in the early autumn compared with the mid-summer time period. There were no significant differences in PC2 scores across time periods in the Illinois or Ohio rivers (Fig. 2b). Variation in PC3 was best explained by the model that consisted of time period only (Table 2). There was significant variation across time periods for PC3 [ANOVA, *F*_(2,_ _495)_ = 14.50, *P* < 0.0001]. *Post hoc* analysis determined that across all rivers, silver carp had the lowest stress scores during the early autumn time period relative to samples collected earlier in the year (Fig. 2c).

## Discussion

Understanding the physiology of invasive species is important to address problems facing conservation and management, and how to control these species (Cooke *et al.*, 2013). Knowledge of nutrition and stress levels can assist in the development of strategies to control the spread of silver carp and help to define predictive relationships between habitat characteristics and individual performance in river basins not yet invaded. For wild-caught silver carp sampled at broad spatial scales, the majority of the variability in the data showed that indices of short-term feeding (PC1; triglycerides, protein, and ALP in plasma) were most strongly influenced by time period, considered independently for each river. Previous research has shown that triglyceride levels in plasma rise following feeding, protein responds to changes in nutritional status (e.g. food consumption, growth, and body condition), and elevated concentrations of ALP are related to the processing of energy substrates by the liver (Wagner and Congleton, 2004; Congleton and Wagner, 2006; Guerreiro *et al.*, 2012). More importantly, all three of these metrics vary according to fasting and refeeding patterns in fishes (Congleton and Wagner, 2006; Hanson *et al.*, 2012). For example, Wagner and Congleton (2004) demonstrated that plasma ALP and protein concentrations declined in juvenile Chinook salmon (*Oncorhynchus tshawytscha*) during fasting, but recovered after feeding was resumed. Hanson and Cooke (2009) also showed that triglyceride values in smallmouth bass (*Micropterus dolomieu*) declined because of fasting during the parental care period of spawning. Mechanisms explaining our observed feeding variability across time periods may include changes in food availability, energetic constraints, or both (Wootton, 1998).

Annually, biotic and abiotic characteristics of rivers can vary significantly. According to Wahl *et al.* (2008), zooplankton densities in the Illinois River peak in late spring, while Burdis and Hoxmeier (2011) observed peak zooplankton densities in May and June in the Upper Mississippi River. This variation in zooplankton data may explain trends in the PC1 scores for silver carp from the Illinois River, because scores were greatest in the mid-summer and late summer, potentially during periods of peak zooplankton concentrations. According to Tyszko *et al.* (2012), silver carp catch per unit effort (CPUE; expressed as number per hour) also peaked in the late summer, which may indicate that feeding is optimal for silver carp in the Illinois River during this time period, regardless of density. Silver carp in the Mississippi River showed consistently low PC1 scores across all three time periods sampled, indicating low feeding rates across all seasons. Despite this uniformity, CPUE of silver carp in the Mississippi River varied across time periods, by as much as 3-fold (Tyszko *et al.*, 2012). This potentially indicates that feeding levels of silver carp are not influenced by density in this river. The complex morphology of rivers has also been shown to vary seasonally in terms of nutrient loading, species composition, and productivity, all of which may influence food availability in the late summer and, in turn, drive PC1 scores (Amoros and Bornette, 2002; Houser and Richardson, 2010). For silver carp in the Midwestern USA, variation in prey abundance may explain observed patters in short-term feeding indices.

Energetic constraints, related to water temperature and/or reproduction, may be another mechanism explaining patterns in short-term feeding (PC1) across time periods. Optimal metabolic conditions, food intake, growth, and nutrient use for fishes are affected by water temperature; fish generally consume more food during periods of elevated temperatures to balance metabolic costs and optimize growth (Kaushik and Médale, 1994; Burel *et al.*, 1996; Guerreiro *et al.*, 2012). Water temperature also affects metabolic rates and maintenance energy requirements for carp, similar to other teleost fishes (Spataru and Gophen, 1985; Kaushik, 1995). For silver carp in the Illinois River, short-term feeding indices were lowest during the early autumn when water temperatures were coolest. In contrast, silver carp in the Ohio and Wabash rivers showed the greatest values for short-term feeding indices during the late summer sampling period, which was the period of highest water temperature for these two locations. Silver carp CPUE was also greatest in the late summer for the Ohio and Wabash rivers (Tyszko *et al.*, 2012), which could suggest that feeding is optimal for silver carp in these rivers during this time period, regardless of density. Reproduction may decrease feeding rates, is metabolically expensive, depletes energy stores (Encina and Granado-Lorencio, 1997), and may result in reductions in short-term feeding indices (Sumpter *et al.*, 1991; Encina and Granado-Lorencio, 1997). Asian carp have been shown to use environmental cues, such as elevated flow, temperature, or river stage, to initiate spawning (DeGrandchamp *et al.*, 2007), with peak larval production typically occurring in the spring (Lohmeyer and Garvey, 2008). Silver carp in the Missouri River, for example, have been observed to reproduce as early as March (Papoulias *et al.*, 2006). Surface velocity was highest across all four rivers during the mid-summer, suggesting that conditions would be suitable for silver carp spawning during this sampling period. This coincides with decreased short-term feeding scores in the Ohio and Wabash rivers during the mid-summer time period. Regardless of the mechanism driving short-term feeding, it was the principal component showing the majority of the variability in silver carp feeding and changed temporally and spatially.

Similar to what was observed for indices of short-term feeding, time period considered independently for each river was the strongest predictor of variation in silver carp body energy reserves (cholesterol and protein in plasma; PC2). Previous studies have shown that plasma protein concentrations respond to changes in body condition, with nutritionally deprived individuals showing reduced protein reserves relative to feeding individuals (Farbridge and Leatherland, 1992). Cholesterol is a lipid present in animal tissue that is positively correlated with body energy reserves (Congleton and Wagner, 2006; Hasler *et al.*, 2011). More importantly, both of these metrics have been shown to vary in response to the feeding history of individual fish. For example, Lambert and Dutil (1997b) showed that protein is an energy reserve that can be mobilized during periods of low energy in Atlantic cod (*Gadus morhua*), while Hanson *et al.* (2012) demonstrated that plasma protein concentrations declined in juvenile Chinook salmon from depletion of energy reserves during smoltification. Congleton and Wagner (2006) also suggested that protein and cholesterol responded to body energy reserves rather than directly to food intake, because these metrics declined slowly in fasted fishes and did not recover after refeeding in juvenile Chinook salmon.

Seasonal differences in food availability and cyclical changes in energy allocation may explain variation in energy stores in silver carp. River temperature can fluctuate greatly, potentially influencing feeding conditions, food availability and quality, and subsequently fish body composition through the allocation of energy resources (Burel *et al.*, 1996; Lambert and Dutil, 1997a, b; Mogensen and Post, 2012). Fishes have the ability to apportion energy to lipid stores to prepare for periods of scarce resources, such as in winter (Mogensen and Post, 2012). Cooler water temperatures in the autumn and winter can reduce activity and feeding rates of fishes (Burel *et al.*, 1996; Johansen *et al.*, 2011), which can minimize energy expenditures and allow for increased energy storage (Morales *et al.*, 2012). Silver carp have also been observed to situate themselves in positions of low water velocity to conserve energy (Calkins *et al.*, 2012). Body energy reserves for silver carp in the Mississippi and Wabash rivers were greatest during early autumn sampling, which was the period of coolest water temperatures recorded for these two rivers. Interestingly, silver carp CPUE rates were also lowest in the early autumn in the Mississippi River (Tyszko *et al.*, 2012). This may contribute to the reason why body condition was greatest, because fewer organisms were competing for resources, allowing silver carp to store energy during this time period in this river. Likewise, Spataru and Gophen (1985) observed that the body condition of silver carp was greatest in autumn and winter relative to other seasons of the year. Analogous to our patterns observed in short-term feeding, high body energy reserves in early autumn may also be related to bioenergetics. The cool water temperatures observed during this time period for all four rivers may have resulted in lower metabolic demands and the absence of energy-consuming spawning events (Burel *et al.*, 1996; Papoulias *et al.*, 2006). Interestingly, body energy reserves of silver carp in the Illinois and Ohio rivers did not differ across time periods despite changes in water temperature of almost 40 and 30%, respectively, during this study. Irrespective of the mechanism(s) changing body energy reserves, our study provides evidence that body energy reserves in silver carp may peak in the early autumn, as observed by the greatest scores in the Mississippi and Wabash rivers during this time period.

Values of PC3 for silver carp (cortisol and glucose in plasma) were highly influenced by time period, independent of river or reach. Given that PC3 was composed exclusively of stress-related variables, our results indicate that stress levels change seasonally, and in synchrony, across broad spatial scales. Plasma corticosteroid (cortisol) is produced during the primary stress response to various physical and psychological stressors for vertebrate animals. Increased plasma glucose levels provide energy as part of the secondary stress response (Barton, 2002; Wagner and Congleton, 2004; Schreck, 2007). Several types of disturbances may trigger the stress response in fishes, including chemical, physical, or perceived stressors (i.e. temperature, nutrition, and water quality; Barton, 2002). For example, season and temperature can exert a strong influence on the hormonal rates of reactions to stress, with higher temperatures causing biological reaction rates to increase (McLeese *et al.*, 1994; Pottinger and Carrick, 2000; Schreck, 2007), and potentially elevating cortisol concentrations (Schreck *et al.*, 2001). Stream-dwelling fishes have been observed to undergo shifts in distribution from summer habitats to overwintering areas to avoid metabolic stress (Schlosser, 1991). Stress levels have also been shown to correlate positively with reproductive activity (Akar, 2011) and negatively with nutritional status (Mommsen *et al.*, 1999). Our results showed that the lowest stress levels in silver carp occurred during the early autumn, regardless of river, which was the period of lowest water temperature for all rivers sampled. Similar results were reported by Jessop *et al.* (2013), who showed that both species-specific and environmental variables were important in reptile and bird responses to stressors across broad scales. Reduced stress levels in silver carp may also be caused by favourable water quality and temperatures (Pottinger and Carrick, 2000), natural, seasonal fluctuations in concentrations of cortisol that are consistent across broad spatial scales, or both (Pickering and Christie, 1981; Koldkjaer *et al.*, 2004). Elevated indices of stress in the mid-summer and late summer may also be correlated with the general timing of silver carp spawning in these rivers (Papoulias *et al.*, 2006; Kolar *et al.*, 2007). Additionally, according to Tyszko *et al.* (2012), CPUE for silver carp was greatest in the late summer in all four rivers sampled, which may have contributed to the higher stress scores. Our results clearly demonstrate that stress in silver carp is driven by time period and is consistent among populations separated across broad spatial scales.

Interestingly, variables previously shown to influence fish nutrition and stress were not significant drivers of variation for silver carp in our study. Principal components describing short-term feeding (PC1), body energy reserves (PC2), and stress (PC3), were not affected by total body length or by reaches within rivers. Previous studies have shown that total length can influence short-term feeding and body energy reserves in fish (Hurst and Conover, 2003; Pangle *et al.*, 2004; Johansen *et al.*, 2011). The capacity for energy storage is often related to body size, where lipid and protein content comprise a greater portion of total body mass in larger individuals (Pangle *et al.*, 2004). In juvenile striped bass (*Morone saxatilis*), lipid reserves were greater in larger individuals, which was attributable to an increase in lipid mass throughout the growing season (Hurst and Conover, 2003). Direct competition between different age and size classes because of diet overlap may also exist, with larger fish having a greater advantage in accessing food, as observed in Atlantic salmon (*Salmo salar*; Johansen *et al.*, 2011). In response to stressors, previous research has also shown that total length and the ontogenetic stage of silver carp can affect the stress response (Schreck, 2007; Laiz-Carrión *et al.*, 2012). Total length may not have contributed to a best fit model because of the number of individuals caught (513) and, even though size varied from 252 to 900 mm, the majority of fish sampled were around the same size (mean 577 ± 6 mm). Our findings are in accordance with those of Encina and Granado-Lorencio (1997), who suggested that seasonal differences in condition, nutrition, and somatic energy content of juvenile and mature *Leuciscus pyrenaicus* were better explained by variation in the environment (e.g. food quantity and quality) than by size.

Likewise, our results also demonstrated that variation in PC scores did not change across reaches within the same river. Previous research has shown that species richness may vary from upstream to downstream locations in rivers, with species richness generally being greater downstream (Karr *et al.*, 1985). Earlier studies on the Illinois River showed significant differences in fish communities across reaches (McClelland *et al.*, 2012), suggesting that reach-scale factors have the potential to impact species assemblages and community structure. However, we did not observe any influence of reach-scale effects on silver carp nutrition and stress. One potential reason may be due to our sampling protocol. Our study was conducted in only 1 year, which was an epic flood year (Olson and Morton, 2012), and this single sampling year might not account for potential inter-annual variability influencing silver carp nutrition and stress. For example, Wagner *et al.* (2010) observed that health indicators of lake whitefish (*Coregonus clupeaformis*) sampled across several locations in Lake Michigan varied across years. Additionally, the rivers we sampled have been greatly altered by anthropogenic influences (i.e. agriculture use, levees, and dams), potentially homogenizing the effects of reach-scale factors at the spatial scales examined.

Despite the findings of our study, there are a number of avenues for future studies to explore related to this topic. For example, the influence of fine-scale habitat features, along with community structure and species diversity and richness, on the health and condition of fishes should be investigated. Examining specific, quantifiable habitat and community characteristics and relating them to nutritional and stress parameters may provide a better comprehension of the link between physiology and invasive species, community structure, and competition, and how interacting factors may influence movement. Other prospective research could address inter-annual variability by continuing similar work over several years for a more comprehensive understanding of silver carp nutritional and stress relationships at a broad spatial and temporal scale, similar to McClelland *et al.* (2012) and Wagner *et al.* (2010). When combined, results from these future studies could help predict the response of Asian carp to habitats not yet invaded and help to predict how Asian carp will perform in different regions should their distributions spread.
